# Mitochondrially targeted p53 or DBD subdomain is superior to wild type p53 in ovarian cancer cells even with strong dominant negative mutant p53

**DOI:** 10.1186/s13048-019-0516-2

**Published:** 2019-05-15

**Authors:** Phong Lu, Erica R. Vander Mause, Katherine E. Redd Bowman, Sarah M. Brown, Lisa Ahne, Carol S. Lim

**Affiliations:** 1 Department of Pharmaceutics and Pharmaceutical Chemistry, University of Utah, 30 S 2000 E Rm 301, Salt Lake City, UT 84112 USA; 2 Philipps-Universitat Marburg, Biegenstraße 10, Marburg, 35037 Germany

**Keywords:** p53, BakMTS, BaxMTS, DBD, Dominant negative, Apoptosis, Mitochondria, Ovarian cancer

## Abstract

**Background:**

While tumor suppressor p53 functions primarily as a transcription factor in the nucleus, cellular stress can cause p53 to translocate to the mitochondria and directly trigger a rapid apoptotic response. We have previously shown that fusing p53 (or its DNA binding domain, DBD, alone) to the mitochondrial targeting signal (MTS) from Bak or Bax can target p53 to the mitochondria and induce apoptosis in gynecological cancer cell lines including cervical cancer cells (HeLa; wt p53), ovarian cancer cells (SKOV-3; p53 267del non-expressing), and breast cancer cells (T47D; L194F p53 mutation). However, p53 with Bak or Bax MTSs have not been previously tested in cancers with strong dominant negative (DN) mutant p53 which are capable of inactivating wt p53 by homo-oligomerization. Since p53-Bak or Bax MTS constructs act as monomers, they are not subject to DN inhibition. For this study, the utility of p53-Bak or p53-Bax MTS constructs was tested for ovarian cancers which are known to have varying p53 statuses, including a strong DN contact mutant p53 (Ovcar-3 cells), a p53 DN structural mutant (Kuramochi cells), and a p53 wild type, low expressing cells (ID8).

**Results:**

Our mitochondrial p53 constructs were tested for their ability to localize to the mitochondria in both mutant non-expressing p53 (Skov-3) and p53 structural mutant (Kuramochi) cell lines using fluorescence microscopy and a nuclear transcriptional activity assay. The apoptotic activity of these mitochondrial constructs was determined using a mitochondrial outer membrane depolarization assay (TMRE), caspase assay, and a late stage cell death assay (7-AAD). We also tested the possibility of using our constructs with paclitaxel, the current standard of care in ovarian cancer treatment. Our data indicates that our mitochondrial p53 constructs are able to effectively localize to the mitochondria in cancer cells with structural mutant p53 and induce apoptosis in many ovarian cancer cell lines with different p53 statuses. These constructs can also be used in combination with paclitaxel for an increased apoptotic effect.

**Conclusions:**

The results suggest that targeting p53 to mitochondria can be a new strategy for ovarian cancer treatment.

**Electronic supplementary material:**

The online version of this article (10.1186/s13048-019-0516-2) contains supplementary material, which is available to authorized users.

## Introduction

High-grade serous carcinoma (HGSC) is the most common and aggressive type of ovarian cancer [1, 2]. It remains the most lethal gynecological malignancy with 30 to 40% overall survival, and the prognosis has not been improved for decades [1–5]. Because of its high heterogeneity with at least 15 implicated oncogenes and 168 epigenetic alterations, targeted therapy for ovarian cancer is difficult [3, 4]. The majority of ovarian cancer patients initially respond well to chemotherapy, but eventually the cancer relapses and develops drug resistance [6, 7]. Therefore, a novel and effective treatment for ovarian cancer is sorely needed. Whole genome sequencing of HGSC samples derived from patients have revealed the key drivers in ovarian cancer. In particular, 96% of all HGSCs have mutation and loss of function of p53 tumor suppressor protein, making p53 gene therapy an attractive approach [3, 4, 6]. However, clinical trials using wild type (wt) p53 gene therapy in ovarian cancer in the late 1990s did not show a therapeutic effect [8]. Dominant negative inhibition, the dimerization and inactivation of exogenous p53 by mutant p53 in cancer cells, was thought to be a contributing factor in p53-wt gene therapy failure [8, 9]. It is also likely that reactivating the traditional tumor suppression activity of p53 was not potent enough to overcome multiple genetic aberrations in cancer cells. Next generation p53 gene therapy should explore alternate pathways to increase efficacy and potency against cancer. We aim to do this by taking advantage of the non-nuclear pathway of p53.

**Fig. 1:**
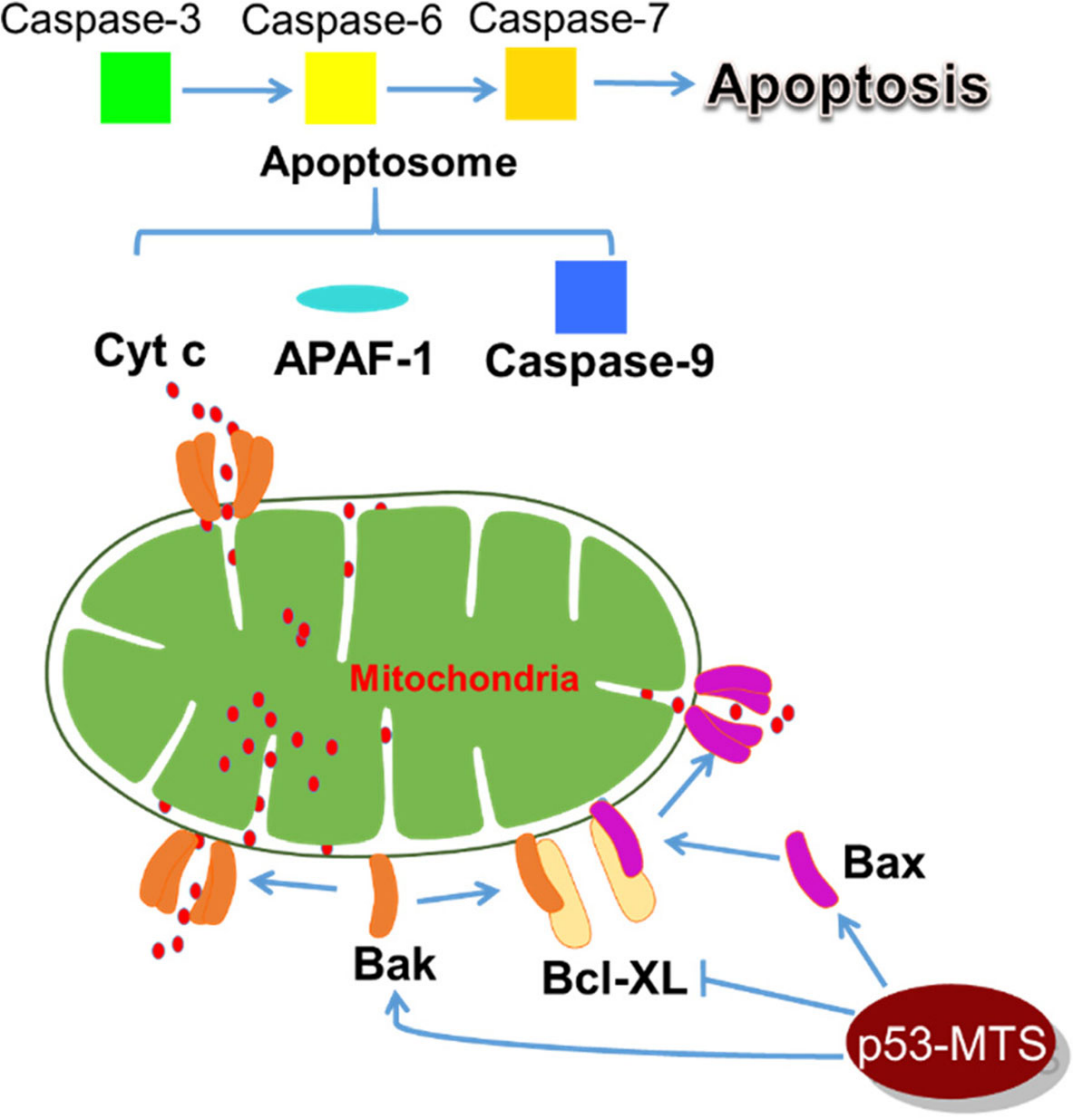
Mitochondrial p53 activates the intrinsic apoptotic pathway. p53 binds and induces the release of Bak (BCL-2 antagonist killer 1) and Bax (BCL-2 associated X protein) from the anti-apoptotic factors [Bcl-2 (B-cell lymphoma 2), Mcl-1 (Induced myeloid leukemia cell differentiation protein), and Bcl-XL (BCL-2 related protein, long isoform)]. The effectors Bak and Bax homo-oligomerize and lead to the formation of permeable pores on the mitochondrial outer membrane. The mitochondrial membrane potential is disrupted, and cytochrome c is released, which leads to the formation of the apoptosome, caspase activation, and ultimately apoptotic cell death

Although the main function of p53 is as a nuclear transcription factor with important roles in cell cycle arrest, DNA repair, and apoptosis, 2–3% of intracellular p53 has been known to localize to the mitochondria upon death signal induction [10–13]. While the exact molecular mechanism for this localization is unknown, the activity of mitochondrial p53 is transcription-independent [10–12, 14]. At the mitochondria, p53 can directly trigger the intrinsic apoptotic pathway through binding and neutralizing mitochondrial anti-apoptotic proteins (Mcl-1, Bcl-2 and Bcl-XL) and activating pro-apoptotic effector proteins (Bak and Bax), which induce the release of Bak and Bax from Bcl-2 and Bcl-XL and trigger the homo-oligomerization of Bak and Bax (Fig. 1) [10–12, 14–17]. This leads to the formation of permeable pores on the mitochondrial outer membrane, which in turn causes the release of cytochrome c and activation of caspase-3 (Fig. 1) [10, 11]. Targeting p53 to the mitochondria is an attractive approach because it can induce a rapid apoptotic response while bypassing the cell cycle arrest pathway. Also, unlike nuclear p53, which only functions as a tetrameric protein, mitochondrial p53 acts as a monomer [9, 18, 19]. Therefore, mitochondrial p53 may overcome the dominant negative effect (a result of tetramerization), a reason that wild type p53 gene therapy has failed. Thus, by avoiding the dominant negative effect and reducing the number of pathways required to reach apoptosis, mitochondrially targeted p53 represents a new, more potent generation of p53 gene therapy.

p53 is not known to have a mitochondrial targeting signal [12], and using p53’s innate mitochondrial localization ability is too weak for therapeutic effectiveness. Rather, active targeting of p53 to the mitochondria using mitochondrial-targeting strategies would be optimal. Previous work in our lab has shown that p53 can be targeted to the mitochondria by fusing the mitochondrial targeting signal (MTS) of pro-apoptotic factors Bak or Bax to the C-terminus of p53 [20–22]. The chimeric constructs are called p53-BakMTS and p53-BaxMTS respectively. It should be emphasized that these p53-BakMTS and p53-BaxMTS fusions only have the MTS domain of Bak and Bax rather than the entire protein. Because p53 is known to directly interact with the effectors Bak and Bax [16, 23], only the MTS domains of Bak and Bax were fused to p53 to avoid unwanted self-interaction. The MTS domains of Bak and Bax are naturally located on the hydrophobic C-terminus of the proteins. The MTS contains a transmembrane (TM) domain that allows insertion of Bak or Bax into the mitochondrial outer membrane [21, 24, 25]. The amino acid sequences of the MTSs of Bak and Bax are GTPTWQTVTIFVAGVLTASLTIWKKMG and GNGPILNVLVVLGVVLLGQFVVRRFFKS, respectively [21]. After p53-BakMTS and p53-BaxMTS are delivered as a gene to the nucleus, the chimeric constructs will be expressed, then transported to the mitochondria. By fusing BakMTS or BaxMTS to the C-terminus of p53, p53 is actively delivered to the mitochondria. p53-BakMTS or p53-BaxMTS can then activate mitochondrial Bak and Bax oligomerization and trigger transcriptional independent apoptosis [21].

Because p53 is known to interact with Bak via amino acids K120, R248, R273, R280, and E287 within the DNA binding domain (DBD) of p53, we showed that the DBD is the minimum domain required for mitochondrial apoptotic effect of p53 [22]. When this DNA binding domain is targeted to mitochondria, it can also induce apoptosis in many cancer cell types [22]. For this reason, we also included both DBD-MTS and p53-MTS fusion constructs in our study.

In this paper, we report the ability of p53-BakMTS and p53-BaxMTS constructs to evade mutant p53 dominant negative inhibition in ovarian cancer cells (including those with a p53 contact mutant and a p53 structural mutant). We also show that mitochondrial p53 constructs are equally effective or superior to wild type p53 in all ovarian cancer cell lines regardless of the p53 status. We also explore the possibility of combining chimeric p53 gene therapy with paclitaxel, the standard of care in ovarian cancer therapy.

## Results

### Localization of the designed chimeric constructs to the mitochondria

**Fig. 2:**
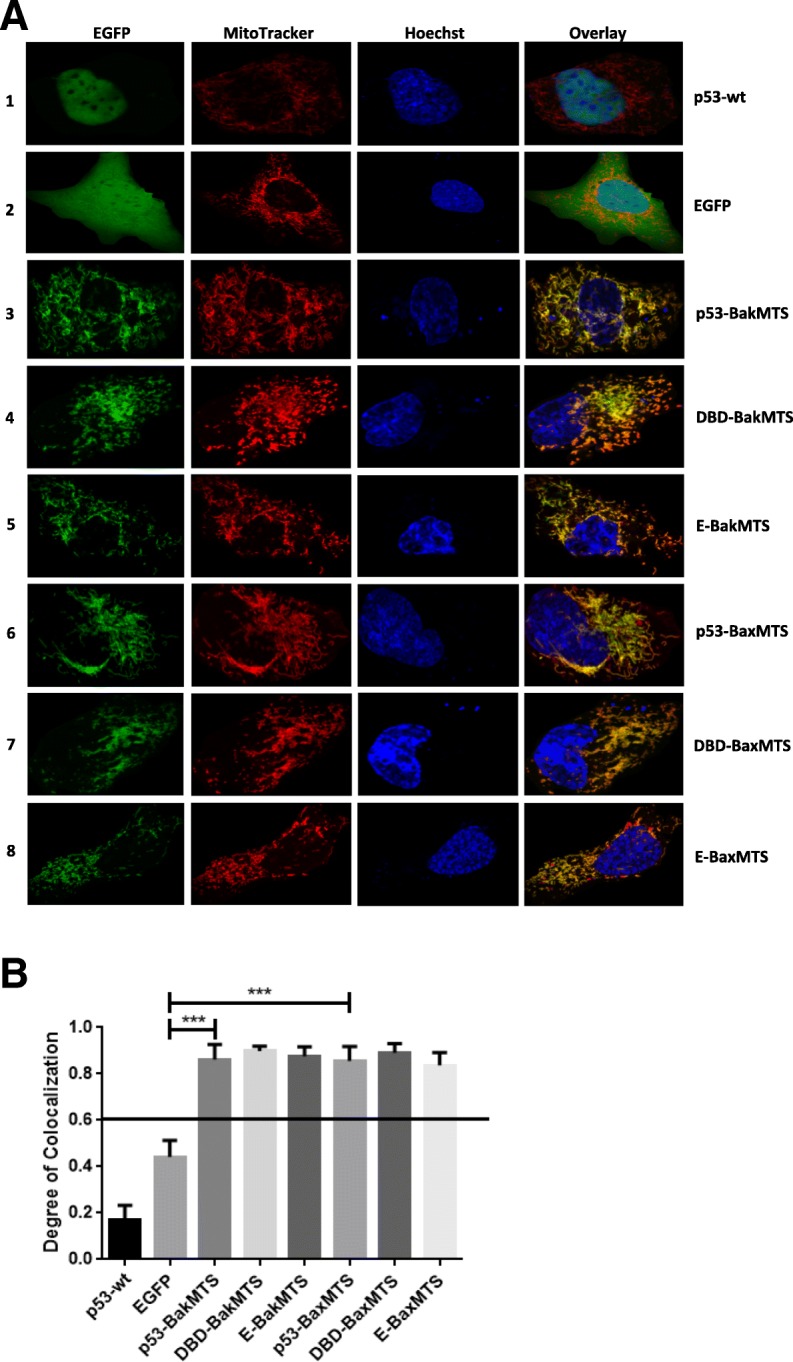
(**a**) Microscopy of EGFP-tagged p53-BakMTS, DBD-BakMTS, E-BakMTS, p53-BaxMTS, DBD-BaxMTS, and E-BaxMTS constructs indicates mitochondrial localization in transfected SKOV-3 cells. The first column (from the left; green color) shows EGFP tagged constructs, the 2nd column (red color) shows stained mitochondria. The 3rd column (blue color) shows the nucleus stained with Hoechst, and the last column shows the overlay. (**b**) The PCC value generated for each individual cell in each construct was graphed. A PCC value of 0.6 and above is considered colocalized with the mitochondria. Statistical analysis was performed using one-way ANOVA with Tukey’s post-test; ***p < 0.001. Error bars represent standard deviations of PCC values from 30 cells per plasmid constructs in 3 independent experiments (*n* = 30). All constructs with Bak or Bax contain only the MTS from those proteins (and not the entire protein)

Since all of the constructs are tagged with EGFP at the N-terminus, fluorescence microscopy was used to track the localization of the constructs after transfection. The mitochondria were stained with MitoTracker Red, and the nuclei were stained with Hoechst. First, we chose the p53 non-expressing cell line SKOV-3 to study the localization of these constructs in cells without endogenous p53 [26]. Figure 2 illustrates the cells transfected with p53-wt, GFP, p53-BakMTS, p53-BaxMTS, the shorter DBD version (DBD-BakMTS, DBD-BaxMTS), and the negative controls with just the MTSs, E-BakMTS and E-BaxMTS.

In SKOV-3 cells transfected with p53-wt, the majority of the exogenous p53 localizes to the nucleus as expected since p53 is a nuclear protein with 3 nuclear import signals (Fig. 2A, row 1, overlay column) [21]. In cells transfected with only EGFP, the protein is found throughout the whole cytosol (Fig. 2A, row 2, overlay column). In contrast, all constructs with the BakMTS and BaxMTS have a clear EGFP signal overlap with the MitoTracker Red whether attached to full length p53 or only the DBD. (Fig. 2A, row 3, 4, 6, and 7, overlay column). The negative control with EGFP attached to BakMTS or BaxMTS (E-BakMTS and E-BaxMTS in Fig. 2A, row 5 and 8, overlay column) also effectively localizes to the mitochondria.

For quantitative comparison of the localization of the EGFP tagged constructs to the mitochondria, the Pearson’s correlation coefficient (PCC) value for each construct was graphed and illustrated in Fig. 1B. A PCC value of 0.6 and above is considered to be colocalized [27]. All of the constructs with Bak or Bax’s MTSs (p53-BakMTS, DBD-BakMTS, E-BakMTS, p53-BaxMTS, DBD-BaxMTS, and E-BaxMTS) show a high degree of colocalization (Fig. 2B, bars 3 to 8). In this non-expressing mutant p53 SKOV-3 cell line, transfected p53-wt localized to the nucleus as expected with the average PCC value of p53-wt of 0.18 (Fig. 2B, bar 1). The EGFP control has an average PCC value of 0.4 (Fig. 2B, bar 2), which also indicates a random distribution. Even though p53-BakMTS and p53-BaxMTS have full length p53’s nuclear import signals, they still effectively localize to the mitochondria, which suggests the ability of the BakMTS and BaxMTS to override the nuclear import signals of p53. Similar results have been observed in ID8 cells, a p53 wild type but low expressing cell line (Additional file 1: Figure S1).

### Mitochondrial p53 evades structural mutation and dominant negative inhibition

**Fig. 3:**
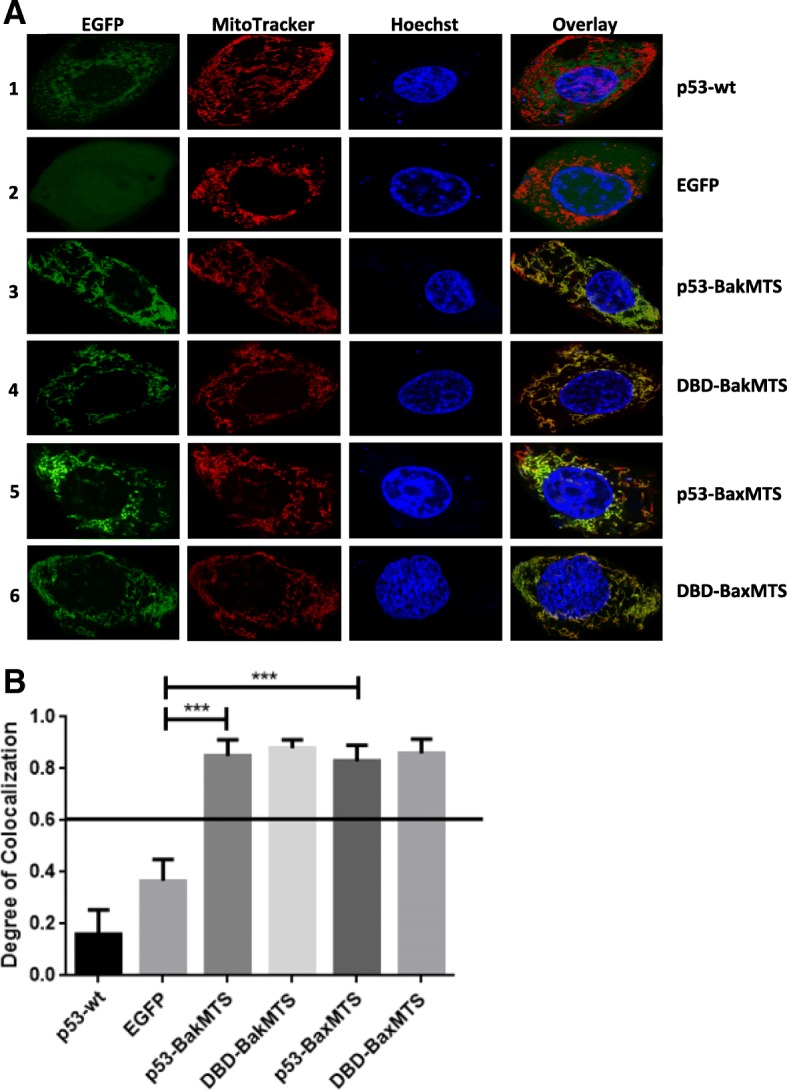
(**a**) Microscopy of EGFP-tagged p53-BakMTS, DBD-BakMTS, p53-BaxMTS, and DBD-BaxMTS constructs indicates mitochondrial localization in transfected Kuramochi cells. The first column (from the left; green color) shows EGFP tagged constructs, the 2nd column (red color) shows stained mitochondria. The 3rd column (blue color) shows the nucleus stained with Hoechst, and the last column shows the overlay of the 3 individual channels. (**b**) The PCC values of each construct were generated and graphed. A PCC value of 0.6 and above is considered colocalized with the mitochondria. Error bars represent standard deviations of PCC values from 30 cells per plasmid constructs in 3 independent experiments (n = 30). ***p < 0.001

After showing that chimeric p53-BakMTS and p53-BaxMTS constructs are able to localize to the mitochondria in a p53 non-expressing cell line, we repeated the experiments in Kuramochi cells, which have a D281Y mutation in the DNA binding domain (DBD) of p53 [28]. The D281Y mutation is thought to cause endogenous p53 to aggregate [29] but has not previously been tested specifically for Kuramochi cells. When Kuramochi cells are transfected with wild type p53, endogenous p53 (supposedly aggregation-prone) binds to exogenous p53, causing the majority of the transfected wild type p53 to accumulate/cluster in the cytoplasm as shown in Fig. 3A (row 1, EGFP and overlay column). Here, we show p53-D281Y mutant clustering in Kuramochi cells, consistent with what is expected for a structural mutant [29, 30]. The distribution pattern of wild type p53 in Kuramochi cells is different from p53 non-expressing SKOV-3 cells (Fig. 3A, row 1 compared Fig. 2A, row 1). However, p53-BakMTS, p53-BaxMTS, DBD-BakMTS, and DBD-BaxMTS still localize effectively to the mitochondria (Fig. 3A, row 3 to 6, overlay column). The PCC values obtained from the chimeric constructs also indicate mitochondrial colocalization (Fig. 3B). Similar to the SKOV-3 cells, the negative control EGFP still exhibits a random distribution throughout the cytoplasm. p53-BakMTS, DBD-BakMTS, p53-BaxMTS, and DBD-BaxMTS show high degrees of colocalization with the mitochondria (PCC > 0.06), while p53-wt exhibits a random distribution. Similar colocalization is observed in OVCAR-3, a human ovarian cancer cell line with the R248Q p53 mutation (a dominant negative DNA contact mutant, which has been previously reported to form p53 aggregates [29]; Additional file 2: Figure S2).

### p53-BakMTS and p53-BaxMTS constructs do not have nuclear activity

**Fig. 4:**
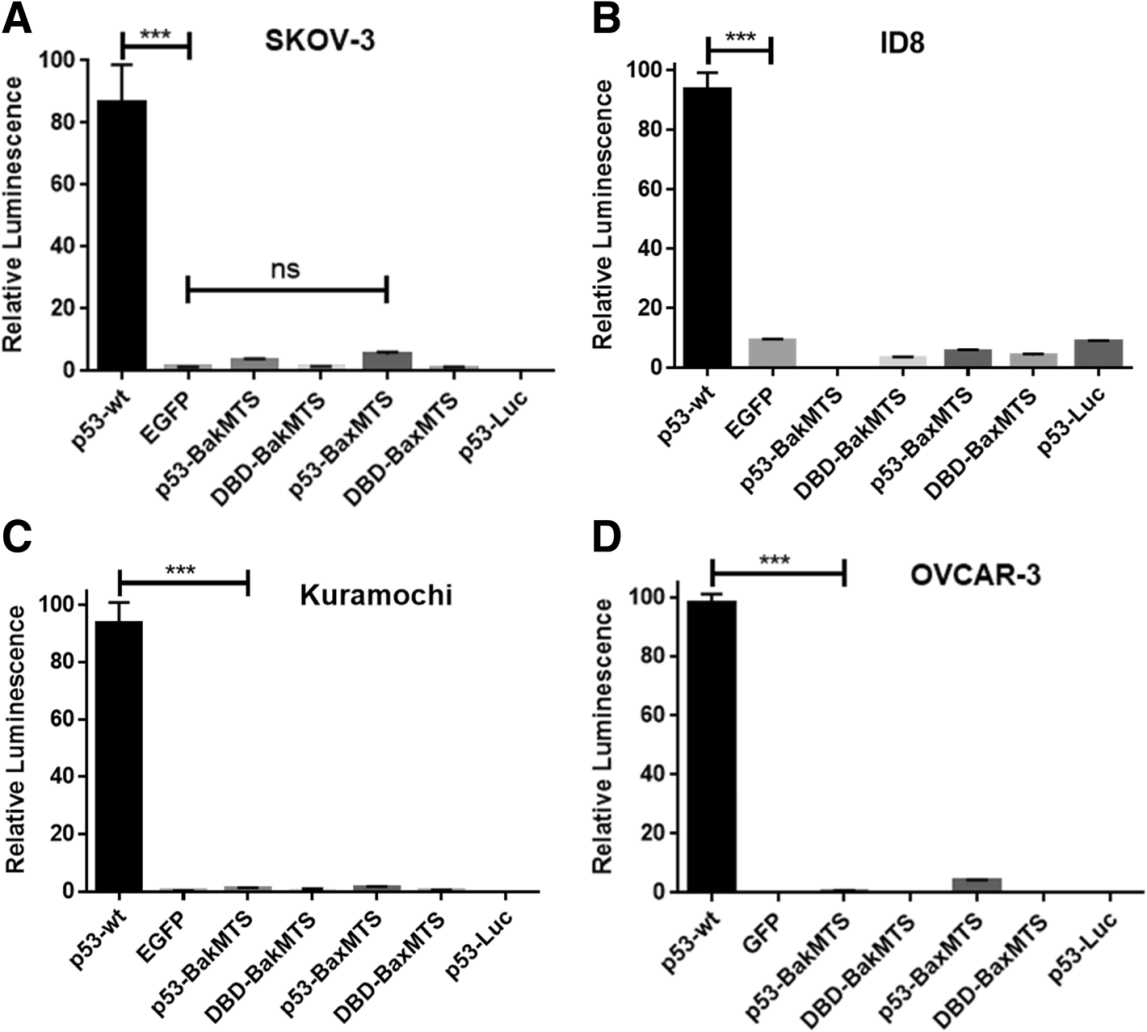
Nuclear transcriptional activity assay was performed to test the ability of all constructs to activate p53-Luc cis-reporter in various ovarian cell lines: (**a**) SKOV-3 has mutant but non-expressing p53; (**b**) ID8 has wild type but very low endogenous p53 expression; (**c**) Kuramochi has dominant negative p53-D281Y mutation; (**d**) OVCAR-3 expresses dominant negative p53-R248Q mutation. p53-wt was used as the positive control; EGFP was the negative control. “p53-Luc only” was the experimental control with only the reporter gene. Error bars represent standard deviations from triplicates (n = 3). ***p < 0.001

To confirm that the activity of p53-BaxMTS and p53-BakMTS is not due to the nuclear transcriptional activity of p53, a p53 reporter gene assay was performed in several ovarian cancer cell lines with different p53 statuses. Each test construct was co-transfected with a firefly luciferase gene under the control of a synthetic promoter, which consists of repeats of the transcription recognition consensus of p53 (TGCCTGGACTTGCCTGG)_14_ [21]. p53-wt is a transcription factor that exerts its tumor suppressor activity mostly through activating pro-apoptotic genes and cell cycle effectors. p53-BakMTS, DBD-BakMTS, p53-BaxMTS, DBD-BaxMTS have no nuclear activity, while p53-wt shows high transcriptional activity. There is no statistically significant difference between the luminescence signals of these designed chimeric constructs and the EGFP negative control (Fig. 4). To confirm that the luminescence signals detected are due to the transfected genes, a control with only the reporter plasmid was included (called p53-Luc). None of the 3 cell lines has endogenous transcriptional activity for p53 (Fig. 4, last bar). Regardless of the status of the endogenous p53, the mitochondrial p53 constructs have no nuclear activity (Fig. 4). Overall, these results indicate that the activity of these MTS constructs does not occur through a nuclear transcriptional pathway. Interestingly, when p53-wt is highly expressed under a CMV promoter in OVCAR-3 (p53-R248Q) and Kuramochi (p53-D281Y) cell lines (which have dominant negative mutant p53), the excess p53 swamps out the dominant negative effect. Therefore, the nuclear transcriptional activity of p53-wt is still observable (Fig. 4B and C, 1^st^ bar). We and others have observed this phenomenon [31].

### p53-BakMTS, p53-BaxMTS, DBD-BakMTS, and DBD-BaxMTS induce late stage cell death

**Fig. 5:**
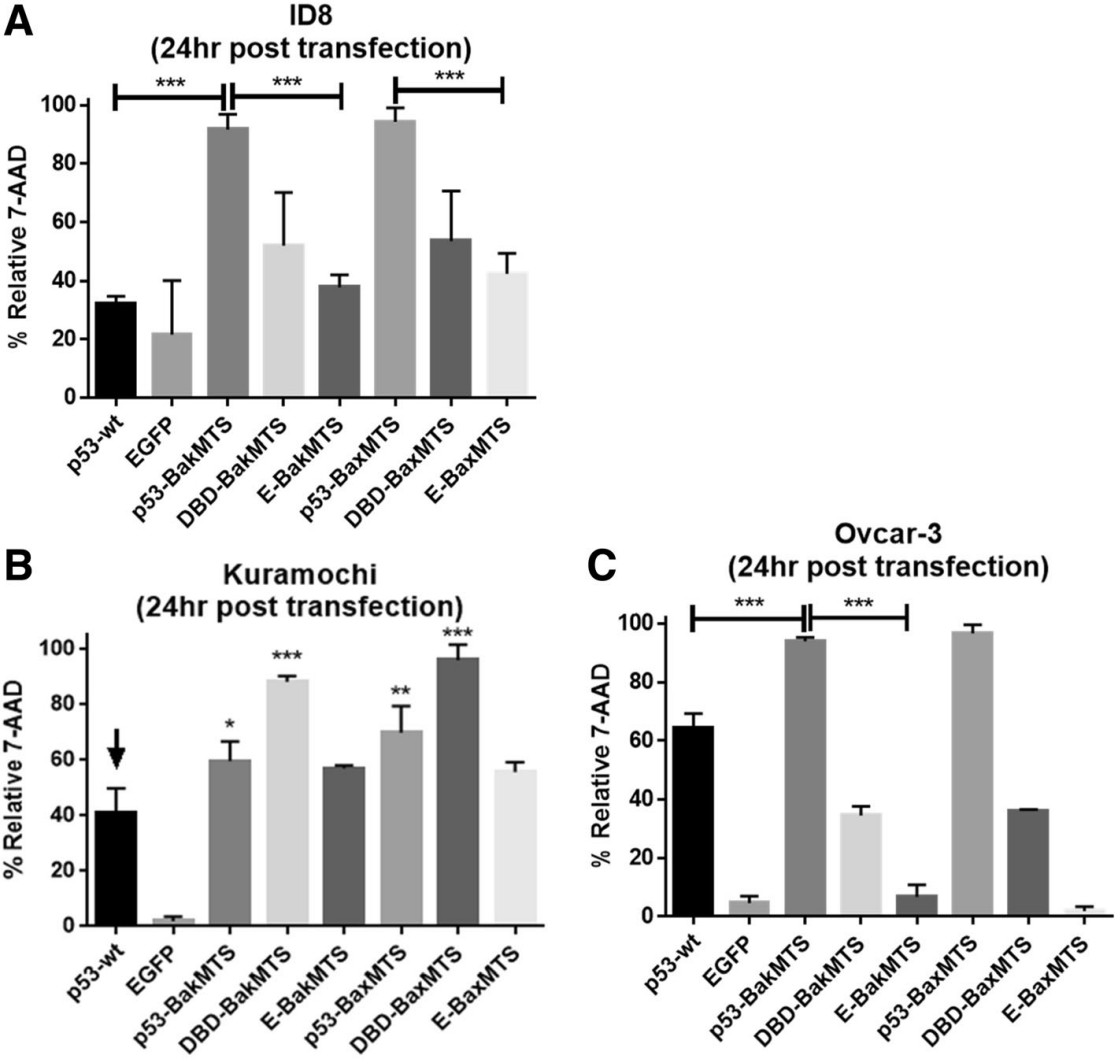
The 7-AAD cell death assay was performed 24 h post transfection in 3 different ovarian cancer cell lines: (**a**) ID8 cells, (**b**) Kuramochi cells, and (**c**) OVCAR-3 cells. Statistical analysis was performed using one-way ANOVA with Tukey’s post-test; *p < 0.05, **p < 0.01, ***p < 0.001 (pairs compared). In Fig. 5B, statistical comparison was performed to compare p53-wt (the arrow mark) and the respective construct. Error bars represent standard deviations from triplicates (n = 3)

After confirming that our mitochondrial p53 constructs effectively localize to the mitochondria and do not have nuclear activity, we tested our constructs’ ability to induce cell death and apoptosis in ovarian cancer cell lines with different p53 statuses. We have previously reported the cell killing activity of our constructs in the non-expressing mutant p53 SKOV-3 ovarian cancer cell line [21]. Both the full length p53-BakMTS, p53-BaxMTS and their respective DBD constructs have superior activity over p53-wt and controls in SKOV-3 as detected by 7-AAD cell death assay. The assay utilizes the DNA intercalating agent 7-AAD, which can only penetrate dying cells with a disrupted cell membrane [21, 32, 33]. Here, 7-AAD were used again to confirm the activity of our constructs in other ovarian cancer cell lines. ID8 has p53-low expression status, while OVCAR-3 has the dominant negative p53-R248Q mutation. In ID8 and OVCAR-3, the full-length p53-BakMTS and p53-BaxMTS have the highest apoptotic activity (Fig. 5A and C). The activity of p53-BakMTS and p53-BaxMTS is significantly higher than p53-wt, EGFP, and negative controls E-BaxMTS and E-BakMTS. p53-wt induces higher apoptosis than EGFP control as expected.

In the Kuramochi cell line, the shorter constructs DBD-BakMTS and DBD-BaxMTS have higher apoptotic activity than the full length p53-BakMTS and p53-BaxMTS (Fig. 5B). The reason for this is unclear, but could be due to a higher degradation rate of full length p53 by MDM2 in Kuramochi cells. MDM2 is the most well-known negative regulator of p53, functioning by tagging p53 for degradation through ubiquitination [34]. The binding of MDM2 to p53 occurs through the N-terminal transactivation domain of p53 [34], which DBD-BakMTS and DBD-BaxMTS lack, thus avoiding MDM2 binding and degradation. However, there are other possible reasons why p53-Bak or Bax MTS may not work as well as the DBD versions in Kuramochi cells, including an antioxidative/survival role of full length p53 (and not DBD) as described in our previous paper [22] or varying levels of MDM2 or MDM2 binding protein (MTBP) involved in stability of p53 [35].

Across all cell lines, high killing activity of these mitochondrial p53 constructs were detected 24 h post-transfection, which may suggest that the mitochondrial p53 constructs are fast acting and rapidly commit the cell to apoptosis and death.

### p53-BakMTS and p53-BaxMTS trigger apoptosis through the intrinsic mitochondrial apoptotic pathway

After confirming the ability of our constructs to induce late stage cell death, we focused our attention to two ovarian cancer cell lines, Kuramochi with dominant negative and structural mutant p53 (D281Y) along with the murine ovarian cancer line ID8 (wild type, low expressing p53) [28, 29, 36]. The mouse ID8 cell line was included because we intend to use the ID8 syngeneic orthotopic tumor animal model in future studies. This model has been shown to closely mimic human ovarian cancer progression in vivo, including the formation of primary tumors, angiogenesis, metastases inside the intraperitoneal cavity, and ascites fluid accumulation [37–39]. On the other hand, Kuramochi is the most representative cell line for high-grade serous carcinoma, and due to its dominant negative and structural mutant characteristics, it is one of the most aggressive and hard to treat ovarian cancers. Moreover, the p53-MTS constructs have not been tested in this type of mutant ovarian cancer cell line [40].

We first investigated whether the activity of our constructs was through the intrinsic mitochondrial apoptotic pathway. There are two major hallmarks of mitochondrial apoptosis – mitochondrial outer membrane permeabilization (MOMP) and caspase cascade activation [19]. To test the ability of p53-BakMTS and p53-BaxMTS to trigger mitochondrial apoptosis, we used the TMRE assay to test the ability of the p53-MTS constructs to depolarize mitochondrial potential. The TMRE assay is a direct measurement of MOMP because homo-oligomerization of the pro-apoptotic factors Bak and Bax triggers outer mitochondrial pore formation, which results in a decreased mitochondrial potential [19].

**Fig. 6:**
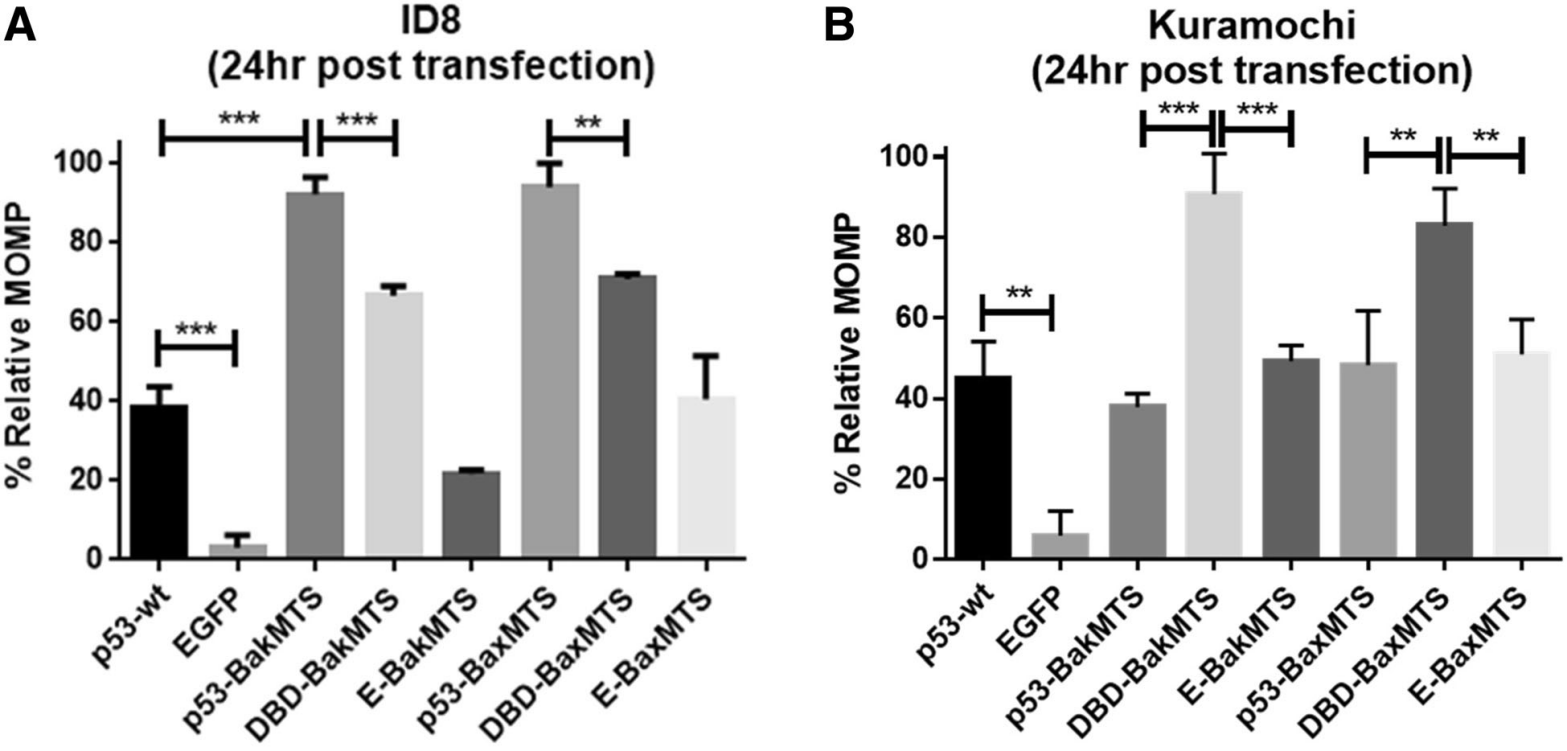
TMRE assay was performed in (**a**) ID8 and (**b**) Kuramochi cell lines (24 h post transfection). TMRE detects mitochondrial outer membrane permeabilization, which occurs as a result of Bak and Bax pore formation, a hallmark of intrinsic apoptosis. Error bars represent standard deviations from triplicates (n = 3). *p < 0.05, **p < 0.01, ***p < 0.001

TMRE is a cell-permeable cationic dye that readily accumulates to active mitochondria due to the highly negative charge found in mitochondria compared to other cytoplasmic compartments [24]. Depolarized mitochondria have decreased membrane potential and fail to sequester the dye. The percent of apoptotic cells can be measured through the loss of TMRE fluorescence intensity by flow cytometry. Percent MOMP is an indicator of early apoptosis. The ability of our constructs to induce MOMP is shown in Fig. 6. In ID8 cells (Fig. 6A), p53-BakMTS and p53-BaxMTS (bar 3 and 6) have superior activity over all other constructs (Fig. 6A). DBD-BakMTS and DBD-BaxMTS also have significant activity over p53-wt (Fig. 6a; compare bars 1, 4 and 7).

In Kuramochi cells with the p53-D281Y mutation (Fig. 6B), the TMRE assay shows no significant difference in activity between p53-wt (bar 1) and the mitochondrial full length p53 constructs (bar 3). However, there is a significant difference in activity between p53-wt and the mitochondrial DBD constructs (bad 1, 4 and 7). This result is consistent with the 7-AAD assay (Fig. 5C). TMRE assay detects the depolarization of the mitochondria, which is the early stage of the intrinsic apoptosis pathway while 7-AAD detects late stage cell death. The consistency across two different assays for both ID8 and Kuramochi suggests that mitochondrial p53-BakMTS, DBD-BakMTS, p53-BaxMTS, and DBD-BaxMTS acts through the mitochondrial pathway.

### Mitochondrial p53 constructs are capable of caspase activation

**Fig. 7:**
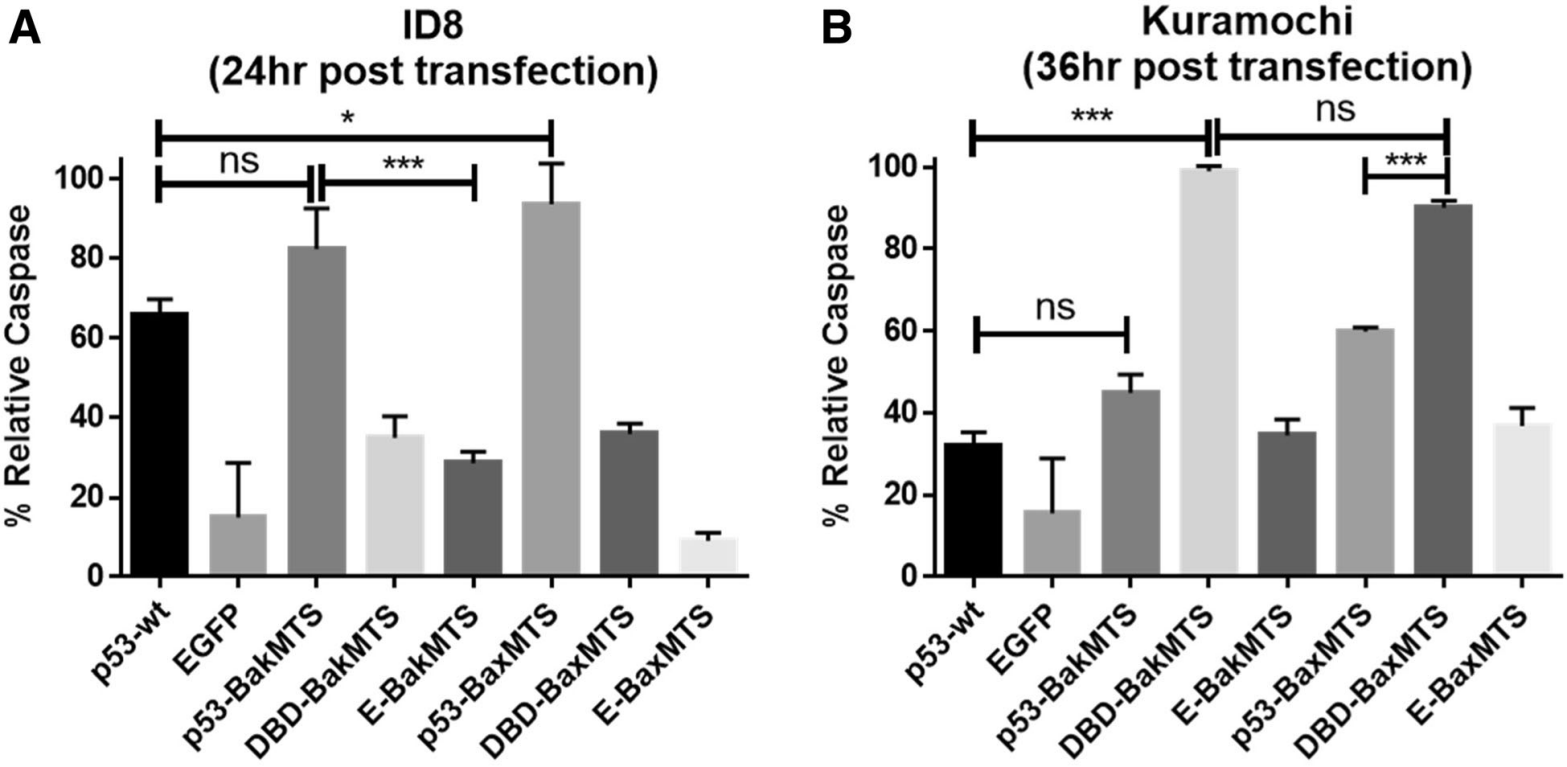
Caspase 3/7 assay was performed in (**a**) ID8 mouse cells (24 h post-transfection) and (**b**) Kuramochi human cells (36 h post-transfection) to measure the percent caspase 3/7 activation with respective constructs. *p < 0.05, ***p < 0.001. Error bars represent standard deviations from triplicates (n = 3)

Caspase activation is the second hallmark of apoptosis. After effectors Bak and Bax homo-oligomerize and help to form pores on the mitochondrial outer membrane, cytochrome c (released from the mitochondria) can bind to Apaf-1 to form the apoptosome, which triggers the caspase cascade and commits the cell to apoptosis. To confirm that the cell killing activity of mitochondrial p53 observed in 7-AAD assay is due to caspase activation, we use caspase 3/7 detection to measure the percentage of the cell population committed to apoptosis. Activated caspase 3/7 was measured 24 h after transfection using a fluorescent dye attached to an inhibitor that specifically binds to active caspase 3/7. The assay measures the percentage of the cell population with active caspase 3 and 7 through flow cytometry detection of the fluorescent dye. In ID8 cells (wild type, low expressing p53), p53-BakMTS, p53-BaxMTS, and p53-wt all have significant caspase activation (Fig. 7A). The negative controls EGFP, E-BakMTS, and E-BaxMTS have minimal activity. This result is also consistent with our previous observation in T47D (L194F p53 mutant) breast cancer cells [21]. For Kuramochi cells, the DBD constructs are the ones with the highest apoptotic activity (Fig. 7B). No significant difference was detected between the two DBD constructs.

### Combination of mitochondrial p53-BakMTS and p53-BaxMTS with paclitaxel

We also attempted to test a combination treatment of our mitochondrial p53 constructs with the small molecule drug paclitaxel, which is a standard of care for ovarian cancer [7]. To determine the IC50 of the drug in ID8 cells, the cell viability MTT assay was performed with a paclitaxel concentration ranging from 0 nM to 500 nM (48 h incubation). The paclitaxel IC50 for ID8 cells was identified to be approximately 30 nM (data not shown). The value is within the reported in vitro IC50 range for ovarian cancer cell lines [41].

**Fig. 8:**
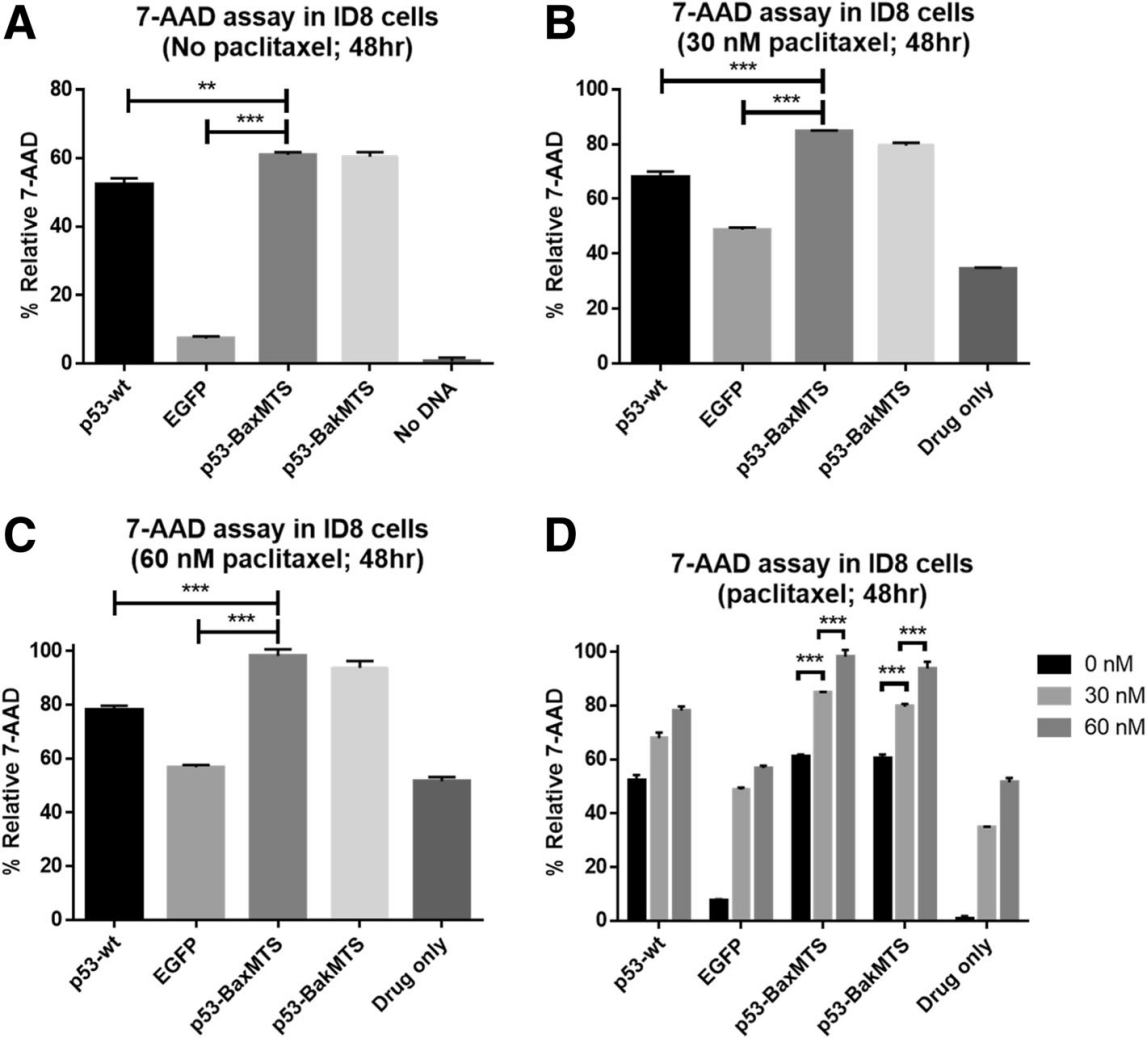
Mitochondrial p53 constructs are tested in combination with paclitaxel in ID8. (A) No drug (transfection only) group was added as the control (no paclitaxel). (**b**) The respective gene construct was used in combination with 30 nM paclitaxel. (**c**) 60 nM paclitaxel was used in combination treatment. (**d**) The apoptotic activities from panel (**a**) to (**c**) are compared across different paclitaxel concentrations. The cells were transfected with the respective gene constructs, and the drugs were added 4 h after transfection at 2 different doses (30 nM and 60 nM). 48 h post-transfection, the cells were collected and analyzed using 7-AAD assay (48 h post-transfection). Error bars represent standard deviations from triplicates (n = 3). **p < 0.01, ***p < 0.001

Since full length p53-BakMTS and p53-BaxMTS are the most effective constructs in ID8 cells in all three different assays (Figs. 5A, 6A, 7A), we only used the full length version of p53-MTS in this combination study. In Fig. 8A, where the cells were only treated with the respective DNA constructs, we see a significant difference between EGFP and p53-BakMTS or p53-BaxMTS as expected (Fig. 8A, compare 3rd bar to 4th and 5th bars). When paclitaxel is added at the IC50 (30 nM) for the ID8 cell line, there is still a significant difference between EGFP and p53-BaxMTS or p53-BakMTS (Fig. 8B). A similar trend is observed when the dose of paclitaxel is doubled to 60 nM (Fig. 8C).

EGFP control with 30 nM of paclitaxel has a significant increase in percent cell death compared to EGFP transfection alone (compare bar 2 of Fig. 8A and B). We routinely observe that the transfection itself makes the cells more “vulnerable” to paclitaxel. When the dose is increased to 60 nM (2 times the IC50), there is a significant difference between EGFP and both p53-BakMTS and p53-BaxMTS (Fig. 8C). This enhanced killing suggests that gene therapy using p53-BakMTS may compliment the cell killing effect of paclitaxel. Additionally, it should also be noted that this 7-AAD assay does not represent the total killing effect of the apoptotic agent. Rather, it only reveals the cell population that is at the late stage cell death at this particular timepoint.

Since paclitaxel takes 48 h for an effect to be seen in vitro, the assays to test the combination of paclitaxel and p53-MTS gene therapy in Fig. 8 were performed at 48 h. At the same time, mitochondrial p53 constructs trigger a rapid apoptotic effect (24 h post-transfection). By testing drugs with different time courses of action, we are likely to “miss” some of the activity of the mitochondrial p53. We also attempted to transfect the cells 24 h after paclitaxel incubation and assayed at 24 h post transfection (48 h paclitaxel incubation), but the paclitaxel had a negative impact on the transfection efficiency (less than 10% of cells were EGFP positive; data not shown).

Therefore, 48 h post transfection/paclitaxel incubation is the earliest time point that the effects of both treatments can be seen, even though the activity of p53-BakMTS and p53-BaxMTS at 48 h may not be at their optimal level (Fig. 5A, 24 h effects are greater since mitochondrial apoptosis induction by our constructs is fast acting). Nevertheless, an increase in apoptosis is seen in the p53-BakMTS with paclitaxel combination groups compared to p53-BakMTS alone (Fig. 8D, bar 4 and 5). The combination of p53-BakMTS and 60 nM paclitaxel also induces significantly higher apoptosis than the combination of p53-wt with the same drug concentration (Fig. 8C and D, compare bar 1, 3 and 4).

**Fig. 9:**
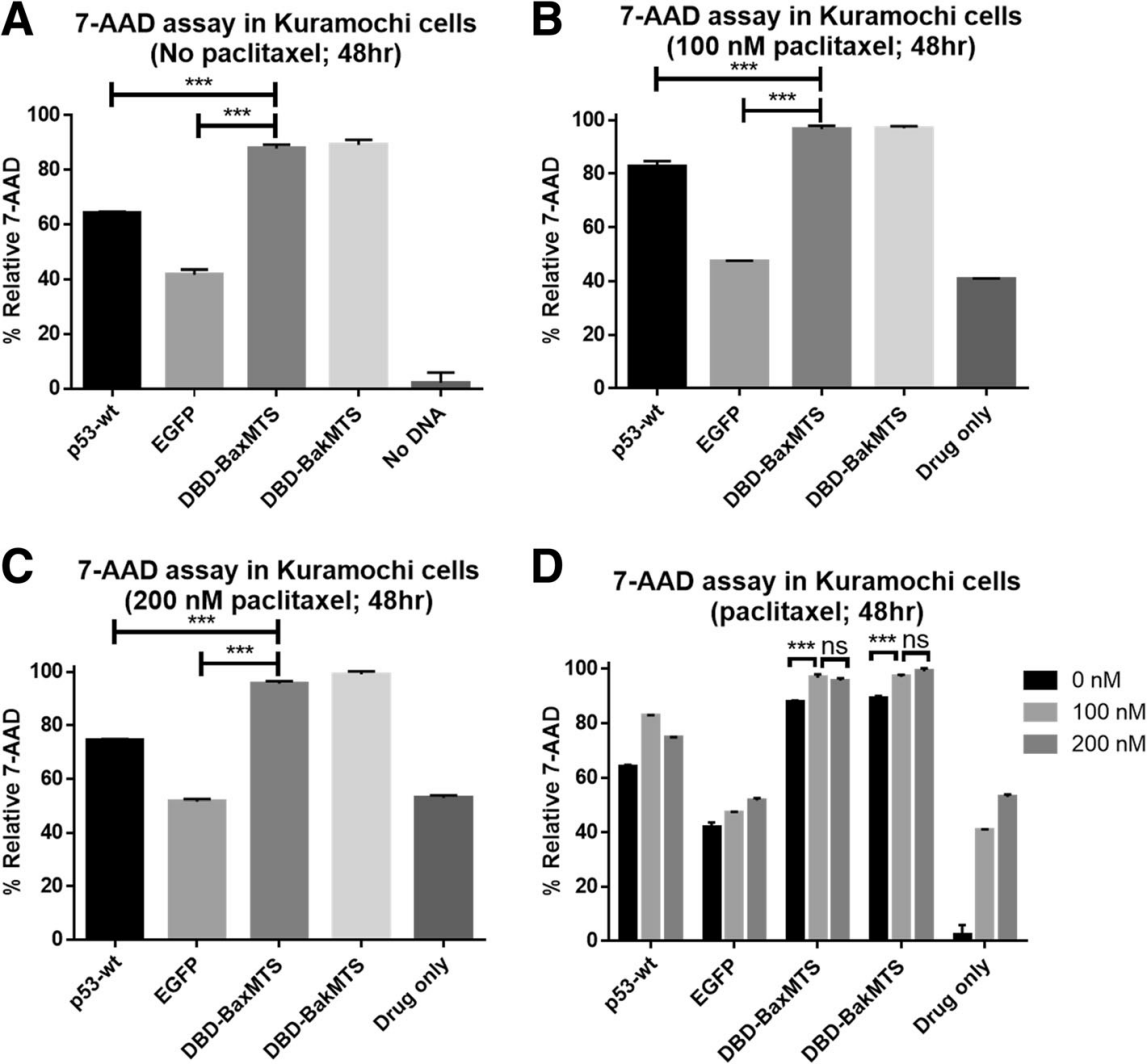
Mitochondrial p53 constructs are tested in combination with paclitaxel in Kuramochi. (**a**) No drug (transfection only) group was added as the control (no paclitaxel). (**b**) The respective gene construct was used in combination with 30 nM paclitaxel. (**c**) (**c**) 60 nM paclitaxel was used in combination treatment. (**d**) The apoptotic activities from panel (**a**) to (**c**) are compared across different paclitaxel concentrations. The cells were transfected with the respective gene constructs, and the drugs were added 4 h after transfection at 2 different doses (100 nM and 200 nM). 48 h post-transfection, the cells were collected and analyzed using 7-AAD assay (48 h post-transfection). Error bars represent standard deviations from triplicates (n = 3). ***p < 0.001

A similar trend is seen for Kuramochi cells. We first determined the optimal paclitaxel concentration for our study. The IC50 for Kuramochi was obtained through the MTT cell viability assay to be 100 nM (48 h incubation). Since DBD-BaxMTS and DBD-BakMTS are the most effective constructs in Kuramochi (see Fig. 5B, 6B, 7B), we only tested the combination of DBD constructs with paclitaxel in comparison with p53-wt and EGFP control. When DBD-BaxMTS and DBD-BakMTS are combined with paclitaxel at IC50 100 nM, there is a signicant increase in cell death activty (Fig. 9D, bar 3 and 4). However, increasing the paclitaxel dose further to 200 nM no longer increases the activity of DBD-MTS constructs in Kuramochi.

## Discussion

Mitochondrial p53 has been known to trigger a direct and rapid apoptotic response [10–13], but little has been done to successfully exploit this transcriptional independent apoptotic pathway of p53 therapeutically. Our strategy is to attach the MTS domains from the mitochondrial pro-apoptotic Bak or Bax proteins to the C-terminus of p53 to actively target p53 to the mitochondria. Once expressed and localized to the mitochondria, p53 can directly activate Bak and Bax to trigger apoptosis [21]. This alternative approach uses p53 to directly induce apoptosis to kill cancer cells instead of simply restoring the normal function of p53. We also showed that the DNA binding domain of p53 is the minimal domain required for the mitochondrial apoptotic activity of p53 [22]. In the past, our lab has also experimented with different MTSs from various mitochondrial proteins including translocase of the outer membrane (TOM), cytochrome c oxidase (CCO), ornithine translocase (OTC), and Bcl-XL to target p53 to the mitochondria [20]. The degree of mitochondrial targeting strongly depends on the strength of the specific MTS and which compartment of the mitochondria (inner membrane, outer membrane, matrix, or intermembrane space) that the MTS targets [20]. Because pro-apoptotic factors and anti-apoptotic factors in the Bcl-2 family are found on the outer mitochondrial membrane, p53 should be targeted to the outer mitochondrial membrane for optimal effects. Additionally, because p53 has three nuclear localization signals (NLS), the chosen MTS must be able to override the three NLSs of p53. BakMTS and BaxMTS are the two strongest and most effective MTS domains that we have tested so far. Here, we show that BakMTS and BaxMTS can direct p53 or DBD to the mitochondria and kill cancer cells with different p53 statuses, even those with dominant negative p53, be it a contact mutant (Figs. 6b) or structural mutant (Figs. 2, 3, and 5).

In terms of missense mutations in cancers, p53 mutations can be broadly classified into two groups – structural mutations and DNA-contact point mutations [42, 43]. DNA-contact mutations such as R248Q (as in OVCAR-3 cells) also exhibit some conformational change compared to p53-wt [29]. Many structural p53 mutations are known to exhibit a myeloid-like characteristic in cancer cells, in which the structural mutant p53 aggregates and exhibits a dominant negative inhibition. Rescuing aggregation caused by structural mutant p53 has been of great interest in p53-based therapies [29, 43, 44]. Many small molecules have been designed to restore the wild type functionality of mutant p53 [44–47]. However, they are mostly specific to certain p53 mutants. For example, the compound NSC319726 is specific to p53-R175H mutation [47]. Another small molecule PRIMA-1, which is currently in clinical trials, was mainly tested in p53-R175H, p53-R273H and p53-R248Q mutations [45, 48]. It is unknown if PRIMA-1 works with any p53 missense mutations. In fact, variable treatment results in Ewing sarcoma have also been reported [49]. Additionally, small molecules designed to restore mutant p53 functionality are expected to have no effect in cancers with wild type p53 or p53 null status [44]. Peptide inhibitors of p53 aggregation such as ReACp53 [29] are also expected to have the same limitations. At the same time, past experience with p53 gene therapy suggests that restoring the regular nuclear function of p53 may not be sufficient in ovarian cancer, which is known to have multiple genetic aberrations [8]. More potent constructs, capable of inducing apoptosis in cancer cells regardless of the p53 status, are needed, such as our mitochondrial targeted p53 constructs. By targeting p53 or its DBD to the mitochondria using BakMTS or BaxMTS, we are able to directly induce apoptosis in Kuramochi cells, which express a p53 structural mutant (Fig. 6B). This is in contrast to p53-wt, which would be captured by endogenous mutant p53 (Fig. 3A, row 1) and have less apoptotic activity compared to our mitochondrial constructs (Fig. 6B). The reason why DBD-BakMTS and DBD-BaxMTS (and not the full length p53-BakMTS and p53-BaxMTS) have the highest cell killing activity in Kuramochi is unclear. One idea is that the lack of the MDM2 binding sites in both DBD-BakMTS and DBD-BaxMTS allow these constructs to escape degradation via MDM2. Full length p53-BakMTS and p53-BaxMTS with an intact MDM2 binding domain are expected targets of MDM2 degradation/inhibition. Another possibility may be due to the difference in full length p53 versus DBD in regulating GPX1, SOD2, ALDH4A1, INP1, TIGAR, Hi95, and PA26 in antioxidative/cell survival pathways, which require the tetramerization domain (TD) and the proline-rich domain (PRD) only found in full length p53 [22, 50-55].

It is thought that structural mutant p53 binds to other p53 molecules via the DNA binding domain to form aggregates [29, 56]. Mutations in amino acid residues 250 to 257 of the DBD are most likely to result in an aggregation mutant phenotype [29, 56]. While the D281Y mutation in Kuramochi cells is not the most common structural mutant, this cell line is thought to be one that most closely mimics human HGSC [40]. The mutation in Kuramochi cells is within the core DNA binding domain and has high aggregation propensity [29]. Particularly, the carboxylate of D281 is known to form a salt bridge interaction with the guanidium side chain of residue R273H. D281Y mutation introduces an aromatic ring that is known to disrupt the loop–sheet–helix motif and the L3 loop of the p53 core domain [30]. It is unlikely that the lower activity of full length p53-BakMTS and p53-BaxMTS as compared to DBD-BakMTS and DBD-BaxMTS in Kuramochi cells are due to p53 aggregation. Because an intact DBD is required for the binding of p53 to proteins Bak and Bax to trigger apoptosis, it is improbable that DBD-Bak or DBD-Bax are interacting with endogenous aggregated cytoplasmic p53.

Our mitochondrial targeting approach may be more advantageous than reactivating p53-wt or rescuing aggregated p53 since restoring traditional nuclear p53 may not be sufficient to kill cancer cells [8, 11]. Mitochondrial p53 bypasses cell cycle arrest and DNA damage repair pathways and thus is more potent than p53-wt [13]. In cancers with p53-wt or p53 deletion, p53-BakMTS and p53-BaxMTS could potentially be used in combination with p53-wt therapy for an additive or synergistic effect because they exploit two different pathways of p53. Specifically, wild type p53 turns on transcription of pro-apoptotic genes like Bax and Noxa for long-term tumor suppression [57, 58], while mitochondrial targeted p53 can directly trigger cell death through the intrinsic apoptosis pathway.

Drug-resistance has been a major challenge in cancer therapy [59]. Ovarian cancer is the most lethal gynecological cancer, and the standard of care, carboplatin and paclitaxel, has remained stagnant for decades [7]. About 80% of women with HGSC will experience tumor progression or a recurrence that is eventually fatal due to the emergence of drug resistance [7]. Letai et al. first introduced the concept of mitochondrial priming regarding drug resistance in cancer cells [60]. Cells with overexpression of anti-apoptotic proteins in the Bcl-2 family such as Bcl-2, Bcl-XL, and Mcl-1 in cancer cells are considered to be “less primed” to apoptosis and are associated with poor prognosis. Ovarian cancer cells are not known to have strong expression of Bcl-2 proteins at the time of diagnosis, and these patients initially respond favorably to chemotherapy with a 50–70% response rate [6, 7, 61, 62]. However, when the cancer becomes drug resistant, 88% of patient samples have strong Bcl-XL expression [61, 62]. These tumors are also more aggressive and resistant to cisplatin, paclitaxel, topotecan, and gemcitabine [57]. Increased Mcl-1 expression is also associated with poor prognosis in HGSC [63]. The importance of the Bcl-2 family as a cancer target has led to the development of many BH3 mimetics that can inhibit Bcl-2 and Bcl-XL. ABT-737 is arguably the best known and well-characterized small molecule that inhibits Bcl-xL and Bcl-2 [63, 64]. ABT-737 and its derivatives induce apoptosis through inhibiting Bcl-2 and Bcl-XL and allowing Bak and Bax activation [64]. However, there is no single small molecule drug that can target all anti-apoptotic proteins. In fact, overexpression of Mcl-1 is a major mechanism of cancer cell resistance to ABT-737 treatment [65–67]. Critically, toxicity has been a major concern for these small molecule drugs [68, 69]. Compared to small molecule inhibitors of Bcl-2 proteins, the ability of mitochondrial p53 to directly activate Bak and Bax is promising especially if coupled with a cancer specific promoter to reduce toxicity. The binding of mitochondrial p53 to Bak disrupts the Bak-Mcl-1 complex and thus counters the anti-apoptotic effect of Mcl-1 [17]. Here we show that actively targeting p53 or DBD to the mitochondria using BakMTS or BaxMTS effectively induces MOMP (Fig. 5), caspase activation (Fig. 7), and late stage apoptosis (Fig. 6) in human ovarian cancer cells that closely mimic HGSC [40]. This activity is observed in many ovarian cancer lines with different p53 statuses. The results suggest that targeting p53 to mitochondria can be a new strategy for ovarian cancer treatment.

We also attempted to combine our mitochondrial p53 constructs with paclitaxel in ID8 and Kuramochi cells. There is evidence that mitochondrial p53 can be combined with paclitaxel for increased efficacy (Fig. 8). We observe a statistically significant combination effect with p53-BakMTS over drug alone or p53-BakMTS alone (Fig. 8D). From our experience, we noticed a time course restriction in our combination study due to the different action times of the 2 drugs (p53-MTS are fast acting constructs and induce MOMP in less than 24 h, while paclitaxel takes a longer time to have an effect). In another experiment, ID8 cells were incubated with paclitaxel for 24 h before transfecting the cells. In that scenario, the efficiency of transfection was very low (less than 10%) (data not shown). In other words, treating the cells with anti-microtubule agents may have a negative impact on the transfection efficiency, which would not be surprising. However, it may be possible to perform consecutive treatments in which the cells are first treated with mitochondrial p53 to maximize the transfection efficiency for optimal apoptotic effect, with small molecule drugs added later to kill off the remaining cancer cells. An animal model may show the true effects of combination therapy. Our next step will be to test the efficacy of our constructs in vivo using a cancer specific promoter coupled with an advanced drug delivery method in a syngeneic orthotopic mouse model that closely mimics human ovarian cancer progression [39].

## Conclusions

p53 or its DBD subdomain can be effectively targeted to mitochondria using MTS from Bak or Bax. This mitochondrial localization is observed even in ovarian cancer cell lines with dominant negative or structural mutant p53. These p53-MTS constructs can efficiently induce apoptosis in many ovarian cancer cell lines with different p53 statuses, and they can also be combined with paclitaxel for an increased apoptotic effect. The results suggest that gene therapy using mitochondrial p53 can be a novel strategy for ovarian cancer treatment.

## Methods

### Cell culture and transient transfection

ID8 murine ovarian carcinoma (a kind gift from Dr. Margit Janát-Asmbury, University of Utah), SKOV-3 human ovarian adenocarcinoma (a kind gift from Dr. Shawn Owen, University of Utah), OVCAR-3 human ovarian adenocarcinoma (ATCC; Manassas, VA), and Kuramochi human ovarian carcinoma (JCRB Cell Bank, Japan) were grown as monolayers in DMEM (ID8, SKOV-3) or RPMI (OVCAR-3, Kuramochi) supplemented with 1% L-glutamine (Thermo Scientific; Waltham, MA), 1% penicillin-streptomycin (Thermo Scientific), and FBS (5% for ID8, 10% for SKOV-3 and Kuramochi, 20% for OVCAR-3). ID8 cells were additionally supplemented with insulin-transferrin-selenium (ITS-X) (Thermo Scientific), and OVCAR-3 cells were supplemented with 0.01 mg/mL bovine insulin (Sigma; St. Louis, MO). For apoptosis assays, 1.5 × 10^5^ cells for ID8**,** 2 × 10^6^ cells for SKOV-3, 3.0 × 10^5^ cells for OVCAR-3 and Kuramochi were seeded in 6-well plates (Corning Life Sciences, Tewksbury, MA). Different numbers of cells were plated to account for varying cell growth rates. 24 h after seeding, transfections were performed using 1 pmol of DNA per well and JetPrime Reagent (PolyPlus Transfection; Illkirch, France) according to the manufacturer’s recommendations. For microscopy, the cells were seeded in 2-well live cell chambers and transfected 24 h after seeding.

### Plasmid constructs

The chimeric gene constructs were cloned as previously described [21]. Briefly, the oligonucleotide sequence encoding the Bak MTS (5′-GGCAATGGTCCCATCCTGAACGTGCTGGTGGTTCTGGGTGTGGTTCTGTTGGGCCAGTTTGTGGTACGAAGATTCTTCAAATCA-3′) or the oligonucleotide sequence encoding the Bax MTS (5′-TCCTACTTTGGGACGCCCACGTGGCAGACCGTGACCATCTTTGTGGCGGGAGTGCTCACCGCCTCACTCACCATCTGGAAGAAGATGGGC-3′) were cloned into the C-terminus of EGFP-p53, EGFP-DBD, or EGFP using BamHI and KpnI restriction sites. The DBD of p53 contains amino acid residues 102 to 292. The cloning of these constructs have been previously described in [22]. The following constructs were used in the experiments: p53-wt, EGFP, p53-BaxMTS, p53-BakMTS, DBD-BaxMTS, DBD-BakMTS, E-BaxMTS, and E-BakMTS (all constructs have EGFP tags).

### Mitochondrial staining and microscopy

24 h post-transfection, cell media was replaced with staining solution containing 200 nM MitoTracker Red CM-H_2_Xros (Thermo Scientific), 1:100 dilution of ProLong Live Antifade Reagent (Thermo Scientific), and 2 μM Hoechst stain in PBS. Cells were incubated for 15 min in the dark prior to imaging. Images were acquired using a Nikon A1R fluorescence confocal microscope with a 60x Plan Apo oil immersion objective, using NIS element software with basic EGFP, MitoTracker, and DAPI filters.

### Image analysis

Images were analyzed for co-localization using the JACoP plugin for ImageJ software [70, 71]. The Pearson’s correlation coefficient (PCC) values were generated with post Costes’ automatic threshold algorithm as before [21]. The PCC value depends on both the pixel intensity and the signal overlap of EGFP and MitoTracker Red [27]. The PCC value has a range from − 1 to + 1 [27]. A PCC value of + 1 indicates complete co-localization, while a PCC value of − 1 indicates anti-correlation [27]. A PCC value of 0 indicates random distribution, and a PCC value equal to 0.6 or above is defined to be co-localized by Bolte and Cordelières [27]. For image analysis, the experiments were performed three times with 10 cells analyzed each time for each individual construct (for a total of 30 individual cells for each plasmid construct).

### Luciferase reporter gene assay

3.5 μg of each individual plasmid construct (p53-wt, EGFP, p53-BaxMTS, p53-BakMTS, DBD-BaxMTS, DBD-BakMTS, E-BaxMTS, and E-BakMTS) were co-transfected with 3.5 μg of p53-Luc Cis-reporter plasmid (Agilent Technologies; Santa Clara, CA) encoding the firefly luciferase gene and 0.35 μg of pRL-SV40 plasmid (Promega; Madison, WI) encoding a Renilla luciferase gene. The pRL-SV40 was used to normalize for transfection efficiency in respective cell lines. The Dual-Glo Luciferase assay system (Promega) was used to detect luminescence signal as previously described [21]. Luminescence was detected 24 h post-transfection using the Infinite M1000 microplate reader (Tecan; Männedorf, Switzerland). The experiment was performed in triplicate (*n* = 3).

### 7-AAD assay

7-AAD cell death assay was performed similarly as previously described [20]. Transfected cells were trypsinized and harvested 24 h post transfection using 0.25% Trypsin EDTA (Thermo Scientific). The suspension was centrifuged for 5 min. at 1000 rpm. The pellet was suspended in 300 μL of 1.25 μg/mL 7-AAD in PBS. Prior to analyzing on the FACS-Canto-II (BD-BioSciences, Franklin Lakes, NJ; University of Utah Core Facility), cells were incubated on ice for 30 min. Cells were gated for proper morphology, EGFP (excitation 488 nm, detection 507 nm), and 7-AAD (excitation 496 nm, detection 785 nm). Each construct was assayed in triplicates (n = 3). The lowest value (EGFP and 7-AAD positive cells) was set as 0%, and the highest value as 100% (relative 7-AAD) as before [21].

### Caspase 3/7 assay

Cells were stained for activated caspase3/7 (ImmunoChemistry Technologies cat no.9125; Bloomington, MN) for analysis via flow cytometry according to the manufacturer’s instructions. Cells were collected as described in the 7-AAD assays. Cell pellets were suspended in 1x apoptosis wash buffer containing FLICA 660 (diluted per manufacturer’s instructions). Samples were incubated at 37 °C for 1 h. Cells were washed 3 times and suspended in 300 μL 1x apoptosis wash buffer prior to analysis by FACS-Canto-II. Cells were gated for morphology and EGFP as in the 7AAD assay. Additionally, cells were gated for FLICA 660 staining (excitation 660 nm, detection at 690 nm). Each construct was assayed in triplicates (n = 3). The lowest value (EGFP and caspase 3/7 positive cells) was set as 0%, and the highest value as 100% (relative caspase 3/7) as before [21].

### TMRE assay

TMRE assay was performed as before [72, 73]. In brief, pelleted cells (collected as described for the 7-AAD assay) were resuspended in 300 uL of annexin-V buffer with 100 nM tetramethylrhodamine ethyl ester (TMRE) from Invitrogen (Carlsbad, CA) 24 h after transfection. Cells were incubated for 30–45 min, then analyzed using the FACS-Canto-II with FACS Diva software. EGFP was excited at 488 nm with emission filter 530/35, and TMRE was excited at 561 nm with the emission filter 585/15. Mitochondrial depolarization (loss in TMRE intensity) correlates with an increase in mitochondrial outer membrane permeabilization (MOMP). Independent transfections of each construct were tested in triplicate three times (n = 3). The lowest MOMP value was set as 0%, and the highest MOMP value as 100% (relative 7-AAD) as previously [21].

### Paclitaxel treatment

Paclitaxel powder (LC Laboratories; Woburn, MA) was dissolved to 10 mg/mL in DMSO according to manufacturer’s recommendation. To determine the IC50 of paclitaxel in ID8 and Kuramochi cells, increasing paclitaxel concentrations were added to appropriate wells with adherent cells in complete growth medium. Each well from the 96-well-plate was seeded with 5000 ID8 or 8000 Kuramochi cells 24 h prior to paclitaxel treatment. 48 h after drug incubation, the Aqueous One Cell Proliferation Assay (Promega) was used to determine cell viability. For transfection and paclitaxel combination treatment, paclitaxel (30 or 60 nM for ID8; 100 nM or 200 nM for Kuramochi) was added 4 h post transfection, and the cells were assayed 48 h after treatment. One-way ANOVA with Tukey’s post-test was used to determine statistical significance as indicated in figure legend.

### Statisical analysis

PrismGraphPad 6 was used to perform all statistical analysis. The data was displayed as mean with standard deviation. One-way ANOVA with Tukey’s post-test was used to determine statistical significance as indicated in the figure legend.

## Additional files

Additional file 1: **Figure S1.** (A) Microscopy of EGFP-tagged p53-BakMTS, DBD-BakMTS, p53- BaxMTS, and DBD-BaxMTS constructs indicates mitochondrial localization in transfected ID8 cells. The first column (from the left; green color) shows EGFP tagged constructs, the 2nd column (red color) shows stained mitochondria. The 3rd column (blue color) shows the nucleus stained with Hoechst, and the last column shows the overlay of the 3 individual channels. (B) The PCC values of each construct were generated and graphed. A PCC value of 0.6 and above is considered colocalized with the mitochondria. Error bars represent standard deviations of PCC values from 30 cells per plasmid constructs in 3 independent experiments (*n*=30). ***p<0.001. (DOCX 1245 kb)

Additional file 2: **Figure S2.** (A) Microscopy of EGFP-tagged p53-BakMTS, DBD-BakMTS, p53-BaxMTS, and DBD-BaxMTS constructs indicates mitochondrial localization in transfected OVCAR-3 cells. The first column (from the left; green color) shows EGFP tagged constructs, the 2nd column (red color) shows stained mitochondria. The 3rd column (blue color) shows the nucleus stained with Hoechst, and the last column shows the overlay of the 3 individual channels. (B) The PCC values of each construct were generated and graphed. A PCC value of 0.6 and above is considered colocalized with the mitochondria. Error bars represent standard deviations of PCC values from 30 cells per plasmid constructs in 3 independent experiments (*n*=30). ***p<0.001. (DOCX 1200 kb)

## Abbreviations

7-AAD: 7-Aminoactinomycin D
DBD: DNA binding domain
DN: dominant negative
HGSC: high grade serous carcinoma
MOMP: mitochondrial outer membrane permeabilization
MTS: mitochondrial targeting signal
TM: transmembrane
TMRE: tetramethylrhodamine ethyl ester
wt: wild type
