# Supplementary material for: Mitochondrially targeted p53 or DBD subdomain is superior to wild type p53 in ovarian cancer cells even with strong dominant negative mutant p53
Source: J Ovarian Res. 2019 May 15;12:45. doi: 10.1186/s13048-019-0516-2 (PMC6521536; doi:10.1186/s13048-019-0516-2)
Supplement: Supplementary file 2 — Figure S2. (A) Microscopy of EGFP-tagged p53-BakMTS, DBD-BakMTS, p53-BaxMTS, and DBD-BaxMTS constructs indicates mitochondrial localization in transfected OVCAR-3 cells. The first column (from the left; green color) shows EGFP tagged constructs, the 2nd column (red color) shows stained mitochondria. The 3rd column (blue color) shows the nucleus stained with Hoechst, and the last column shows the overlay of the 3 individual channels. (B) The PCC values of each construct were generated and graphed. A PCC value of 0.6 and above is considered colocalized with the mitochondria. Error bars represent standard deviations of PCC values from 30 cells per plasmid constructs in 3 independent experiments (n=30). ***p<0.001. (DOCX 1200 kb) [file 13048_2019_516_MOESM2_ESM.docx]

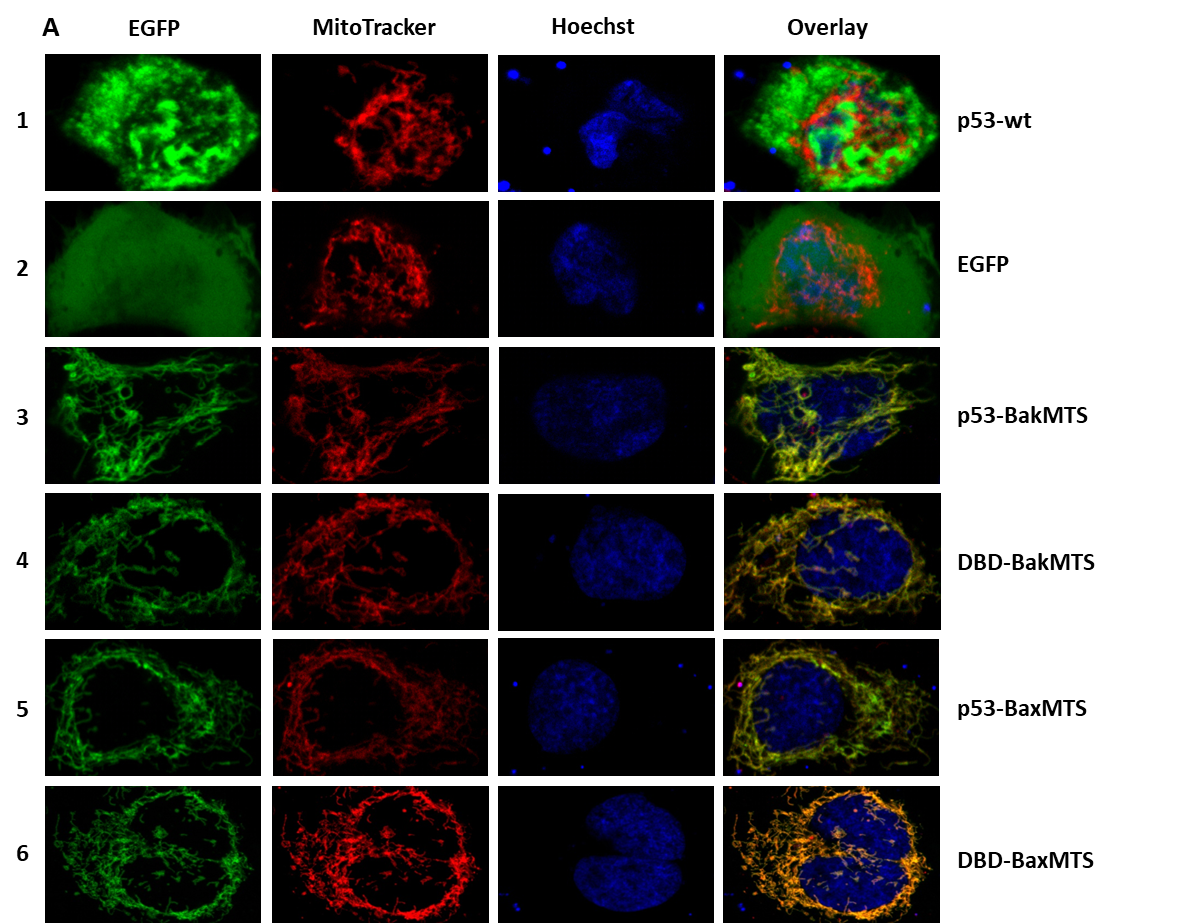


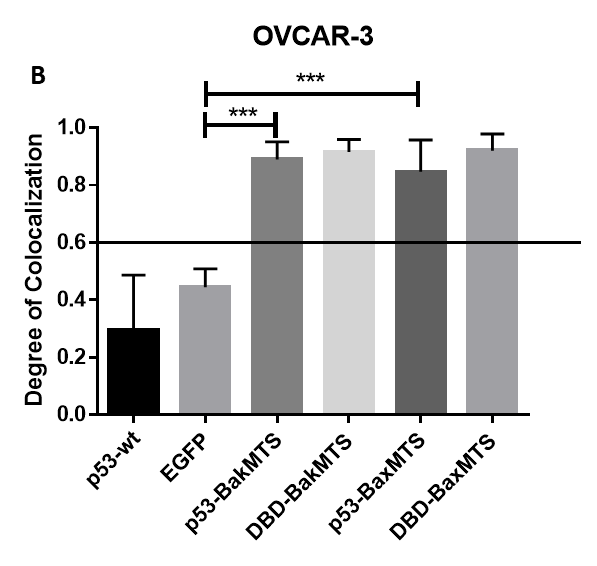


Supplemental figure 2. (A) Microscopy of EGFP-tagged p53-BakMTS, DBD-BakMTS, p53-BaxMTS, and DBD-BaxMTS constructs indicates mitochondrial localization in transfected OVCAR-3 cells. The first column (from the left; green color) shows EGFP tagged constructs, the 2nd column (red color) shows stained mitochondria. The 3rd column (blue color) shows the nucleus stained with Hoechst, and the last column shows the overlay of the 3 individual channels. (B) The PCC values of each construct were generated and graphed. A PCC value of 0.6 and above is considered colocalized with the mitochondria. Error bars represent standard deviations of PCC values from 30 cells per plasmid constructs in 3 independent experiments (n=30). ***p<0.001.
